# Dexamethasone in preventive analgesia alleviates pain and complications after jaw cyst enucleation: a randomized controlled trial

**DOI:** 10.1186/s12871-022-01895-z

**Published:** 2022-11-11

**Authors:** Wang Zhou, Fan Liu, Junbiao Fang, Lianghui Han

**Affiliations:** 1 Center for Rehabilitation Medicine, Department of Anesthesiology, Zhejiang Provincial People’s Hospital, Affiliated People’s Hospital, Hangzhou Medical College, NO.158 Shangtang Road, Hangzhou, Zhejiang 310014 People’s Republic of China; 2 Department of Ophthalmology, Changxing People’s Hospital of Chongming District, No.1008 Fengfu Road, Shanghai, 201913 People’s Republic of China

**Keywords:** Dexamethasone, Jaw cysts, Anesthesia and analgesia, Edema, Trismus

## Abstract

**Background:**

Dexamethasone is widely used in the prevention of postoperative complications in oral surgery and strengthening the analgesic effect after anesthesia, but the efficacy is controversial, and the relationship between postoperative complications and pain is still unclear. The purpose of this study was to evaluate the analgesic effect of dexamethasone in the treatment of jaw cyst and to explore the relationship between postoperative complications and pain.

**Methods:**

We conducted a prospective, randomized, double-blind clinical trial. 120 patients were divided into two groups, dexamethasone group ( group D) and control group (Group C). All patients were given 0.02 mg·kg^−1^ of hydromorphone to relieve pain in advance at 10 min before the beginning of operation. Meanwhile, dexamethasone was injected 0.2 mg·kg^−1^ intravenously in group D and normal saline was injected in group C. The primary endpoint was pain intensity at 2 h, 6 h, 12 h, 24 h and 48 h after surgery. The secondary endpoints were the incidence and extent of complications after surgery, including facial swelling and trismus.

**Results:**

Compared with group C, the visual analogue scale (VAS) scores and occurrence of painful event postoperatively in group D were significantly lower both at rest (*P* < 0.0001 and *P* = 0.0014) and during mobilization (*P* < 0.0001 both). The degree of facial swelling and trismus in group D were significantly lower than that in group C at 24 h (*P* < 0.0001 and *P* = 0.00022) and 48 h (*P* < 0.0001 and *P* = 0.00015) after surgery, but there was no difference at 6 h and 12 h (*P* = 0.137 and *P* = 0.083) after surgery. The C-reactive protein (CRP) level at 24 h after operation in group D was lower than group C (*P* = 0.012), but there was no significant difference in blood glucose concentration between the two groups (*P* = 0.608).

**Conclusion:**

Dexamethasone can reduce the degree of facial swelling and trismus after jaw cyst surgery by inhibiting the production of inflammation, which alleviated the postoperative pain of patients significantly. In addition, it did not increase the risk of hyperglycemia.

**Trial registration:**

This study was registered with the Chinese Clinical Trial Registry on May 07, 2020 (URL: http://www.chictr.org.cn/showproj.aspx?proj=53344. Registry number: ChiCTR2000032693). Registered on 07/05/2020.

## Background

Enucleation of jaw cyst is one of the most common oral surgery operations [1]. Due to the need to damage the soft tissues of the oral cavity and remove bone tissue, there is a strong degree of postoperative inflammation, often accompanied by moderate or severe pain, oedema and trismus, which increases the pain and discomfort of patients [2]. The demand for a comfortable postoperative recovery and a rapid return to daily activities has increased the importance of controlling postoperative inflammation, especially pain and swelling, which also make oral surgery be considered a good model and the gold standard in pain studies [3].

To alleviate the associated postoperative acute pain and the risk of transforming chronic pain in the long term, preventive analgesia methods are widely used in clinical anesthesia [4, 5]. In oral surgery, dexamethasone is often used to prevent postoperative complications [6], while in anaesthesia it is also commonly used as an adjunct to anaesthetic analgesia, to enhance the effect and duration of postoperative analgesia and to reduce the use of postoperative analgesic drugs [7, 8]. However, the efficacy is controversial and uncertain, and even fewer studies have reported on the relationship between postoperative complications and pain. We were interested in knowing whether the facial swelling and trismus after oral surgery were directly proportional to postoperative pain, therefore, this study will evaluate the efficacy of dexamethasone in postoperative analgesia and prevention of postoperative complications in jaw cyst surgery and explore the relationship between the facts.

## Methods

### Ethics approval, registration and patient selection

This is a prospective, randomised, double-blind study taking place at Nantong University Hospital and Zhejiang Provincial People's Hospital from May 2020 to April 2021. The study was approved by the Ethics Committee of the Affiliated Hospital of Nantong University (ethical approval number: 2019-K094) and registration was completed with the China Clinical Trials Centre (registration number: ChiCTR2000032693). Written informed consent was obtained from all participants before the conduct of this study. This trial was conducted in accordance with the Declaration of Helsinki. And this manuscript adheres to the Consolidated Standards of Reporting Trials (CONSORT) guidelines.

A total of 120 American Society of Anesthesiologists (ASA) Class I-II surgical patients between the ages of 16 and 65 years were recruited for this study, and the procedure was performed under general anesthesia with nasal intubation for maxillary cyst excision, and the maxillary cysts were all less than 5 cm in diameter. Participants without recent hepatic or renal insufficiency, severe allergic or hypersensitivity reactions to relevant drugs, cardiovascular or neurological disease, pregnant women or patients with airway difficulties, obesity and those taking opioids were excluded. We randomised 120 patients into groups D (dexamethasone group) and C (control group) using a random number table and the results of the randomisation grouping were sealed in opaque envelopes until the pretreatment drugs were prepared. Neither the patients nor the anaesthetists involved in the study were aware of the results of the random grouping.

### Study procedures

All patients did not receive any preoperative treatment and were monitored in the operating room for non-invasive blood pressure (BP), Electrocardiogram (ECG), peripheral oxygen saturation with Surgical Pleth Index (SPI) and electroencephalographic bispectral index (BIS). Each patient was induced with sufentanil 0.3 µg·kg^−1^, propofol 2–2.5 mg·kg^−1^ and cis-atracurium 0.2 mg·kg^−1^. Anesthesia was maintained by a combination of intravenous and inhalation methods, 1.0% sevoflurane by inhalation in all patients, and intravenous infusion of remifentanil 6–12 µg·(kg·h)^−1^ and isoproterenol 3–5 mg·(kg·h)^−1^, adjusted according to hemodynamic parameters which fluctuated around 20% of basal values of blood pressure and heart rate that the basic value was the average value of blood pressure and heart rate measured three times (5 min apart every time) in a calm state after the patient entered the operating room. During surgery, PetCO_2_, BIS and SPI values were maintained at 35–45 mmHg (1 mmHg = 0.133 kPa), 40–55 and 30–50 respectively. We adjusted the speed of propofol at 0.5 mg·kg^−1^ each time if BIS values were out of our target range and the speed of remifentanil at 1 µg·kg^−1^ each time if SPI values were out of target range, if BIS and SPI values were within normal range but hemodynamic parameters were below or beyond than 20% of the basic values, we adjusted BP by intravenous Norepinephrine 20 µg each time or Urapidil Hydrochloride 5 mg each time and HR by intravenous Atropine 0.5 mg each time or Esmolol Hydrochloride 10 mg each time to our target range with repeated injection. Ten minutes before the start of operation, all patients were given intravenous hydromorphone 0.02 mg·kg^−1^ to anticipate postoperative analgesia, meanwhile, patients in group D received intravenous dexamethasone 0.2 mg·kg^−1^ and patients in group C received intravenous equal doses of saline.

### Outcome measures

The primary indicators for this study were to evaluate the pain intensity and occurrence of painful event, including the resting pain and active pain in the 48 h postoperative period. Secondary indicators included assessing the facial swelling and restricted mouth opening of the patients in the 48 h postoperative period, and monitoring the patients' adverse effects, such as changes in blood glucose. Another anaesthetist, who was unaware of the intervention, performed the outcome assessment.

#### VAS (Visual Analogue Scale) pain scale

A 10 cm horizontal line is drawn across the top of the paper, with 0 at one end of the line indicating no pain, 10 at the other end indicating extreme pain, and the middle section indicating varying degrees of pain. The patient is asked to mark a mark on the horizontal line to indicate the degree of pain according to his or her self-perception, and the length is measured. In clinical practice, it was called moderate and severe pain when we measured the patient's VAS score > 3. And in our study, we defined the patient as having a painful event if the score above 3 points after surgery, we calculated for each patient the occurrence of painful event throughout the repeated measurements at 2 h, 6 h, 12 h, 24 h and 48 h postoperatively.

#### Facial swelling grading

**Fig. 1:**
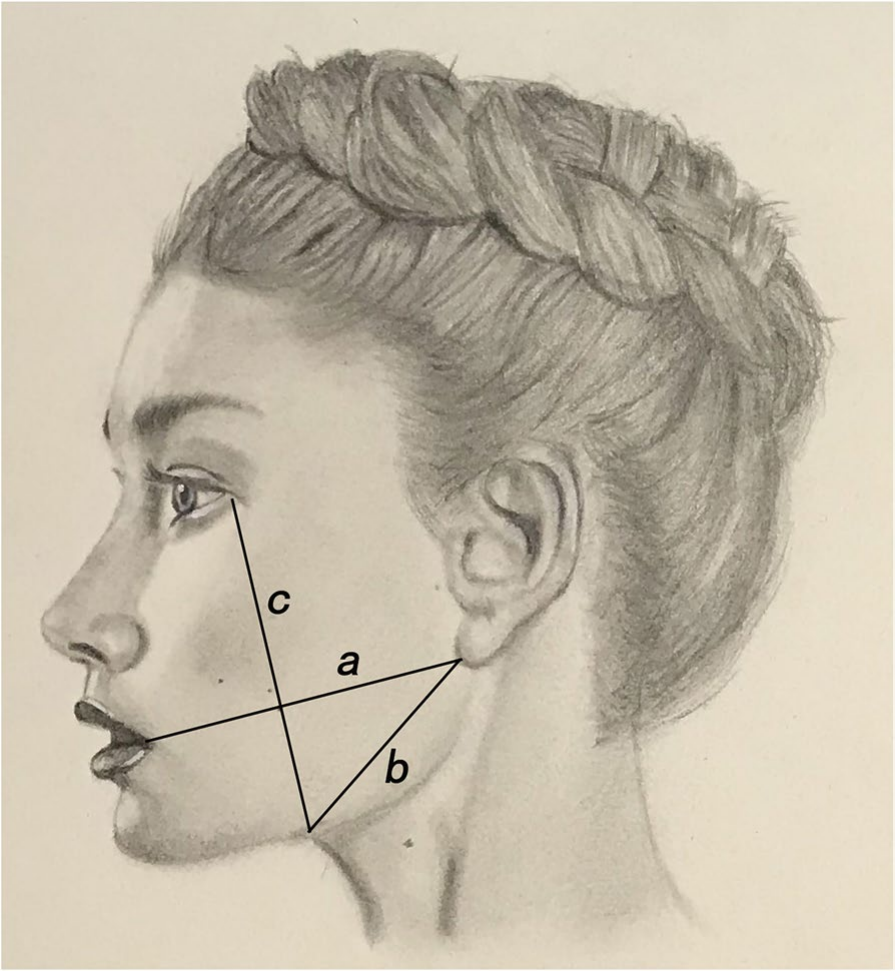
Diagram of facial swelling measurement. **a** The distance from the corner of the mouth to the earlobe on the extraction side. **b** The distance from the earlobe to the mandibular angle. **c** The distance from the external canthus to the mandibular angle. Calculating the facial measurement distance (X) according to formula X = [(a + b)/2 + c]/2, the X value was taken as the average value of facial measurements in millimeters in three times. And then calculating the facial swelling percentage = [postoperative facial measurement distance (X1)—preoperative facial measurement distance (X0)] / preoperative facial measurement distance (X0) * 100%

Reference and improvement of Daniel Lim [9] study method, specific approach: first measure the distance from the corner of the mouth to the earlobe on the extraction side (a), the distance from the earlobe to the mandibular angle (b) and the distance from the external canthus to the mandibular angle (c) respectively (Fig. 1), calculate the facial measurement distance X = [(a + b)/2 + c]/2, and then calculate the facial swelling percentage, the calculation formula is [postoperative facial measurement distance (X1)—preoperative facial measurement distance (X0)] / preoperative facial measurement distance (X0)*100%. The facial swelling was assessed according to the facial swelling percentage, and the criteria: Grade 0, swelling area < 3%; Grade I, swelling area 3 to 6%; Grade II, swelling area 6 to 12%; Grade III, swelling area > 12%. In statistics, we considered Grade 0 and Grade I as light facial swelling while Grade II and Grade III as heavy.

The facial swelling was assessed according to the facial swelling percentage, and the criteria were: Grade 0, swelling area < 3%; Grade I, swelling area 3 to 6%; Grade II, swelling area 6 to 12%; Grade III, swelling area > 12%. we considered Grade 0 and Grade I as light facial swelling while Grade II and Grade III as heavy.

#### Grading criteria for trismus

The distance between the incisal margins of the maxillary and mandibular central incisors is measured with vernier calipers. Grade 0, spacing > 2.5 cm; Grade I spacing 2–2.5 cm; Grade II, spacing 1–2 cm; Grade III, spacing < 1 cm. In statistics, we considered Grade 0 and Grade I as light trismus while Grade II and Grade III as heavy.

### Sample size

The sample size estimation was based according to the active pain intensity 24 h after operation. Our preliminary study found that the mean VAS of the group C and the group D were 4.55 ± 0.98 and 3.90 ± 0.74 (10 patients each group). A power analysis was done with the use of G* Power 3.1.9.7 software and an effect size of d = 0.75 was calculated. A sample size of 48 per group to achieve a power of 95% and a type I error of 5%. To compensate for the possibility of dropout, we eventually recruited a total of 120 patients.

### Statistical analyses

All statistical analyses were performed with SPSS version 20. The Shapiro–Wilk test was applied to assess the normality of the data. To verify the homogeneity of variance, a Levene test was conducted. Quantitative variables with Gaussian distribution were presented as the mean ± standard deviation (SD) or mean with 95% confidence intervals and nonparametric data as a median and interquartile range (IQR). The statistical significance of differences between groups was analysed using the independent t-test to the variables with Gaussian distribution, and analysis of Mann–Whitney U test was used to analyse the non-parametric values. We conducted a linear mixed model (LMM) analysis to compare the difference of postoperative pain between two groups as we performed repeated measurements at different time points postoperatively. Occurrence of painful event was performed with a median with range by Graphpad prism 6.0 and analysed by Mann–Whitney U test. Categorical variables were expressed as number (proportion) and analysed by Pearson χ^2^ test or Fisher exact test, such as ASA classifications, gender, facial swelling and mouth opening level. All figures were plotted with Graphpad prism 6.0 statistical software. A statistically significant difference was determined at a *P* value < 0.05.

## Results

### Patient characteristics

**Fig. 2:**
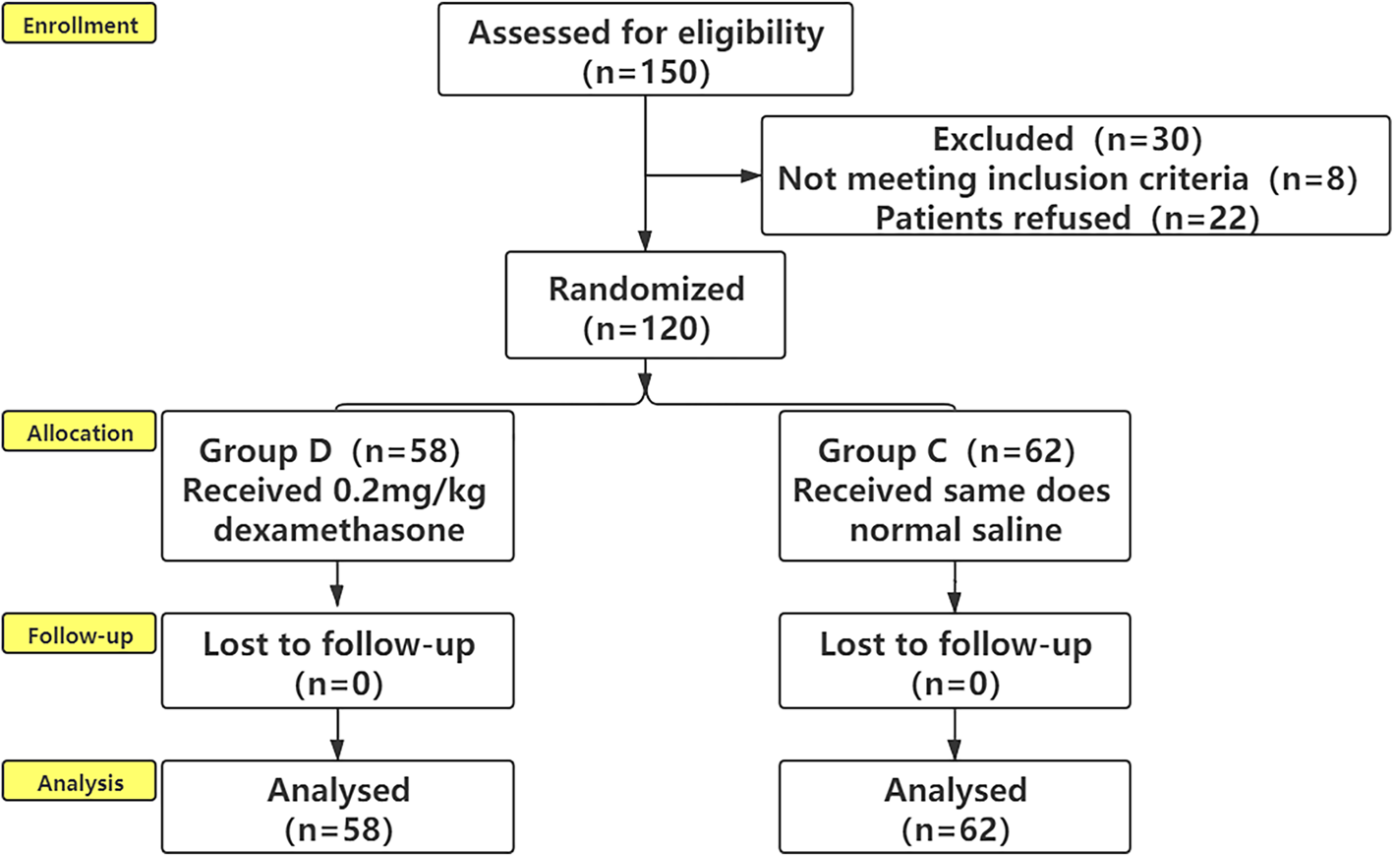
CONSORT diagram of patients recruitment. Flow diagram describing the 150 (120) subjects who completed the study protocol

A total of 150 patients were recruited for this study. However, 30 patients were excluded due to failure to meet the inclusion criteria or patient refusal (Fig. 2). The demographic data and ASA status were compared among the two groups. We found no statistically significant differences in the demographic data among the two groups (*P* > 0.05, Table 1).

**Table 1:** Demographic data and surgery-related information

Parameter	Group D (*n* = 58)	Group C (*n* = 62)	*P*-value
Gender; M/F (cases)	29/29	28/34	0.596
Age (years)	43.5 (26.8—55.3)	42.0 (27.8—55.0)	0.962
Weight (kg)	67.3 ± 8.81	69.0 ± 7.43	0.270
BMI (kg/m^2^)	21.9 ± 2.59	21.6 ± 2.32	0.583
ASA status (I/II)	46/12	51/11	0.682
Duration of surgery (min)	40.2 ± 9.62	41.5 ± 9.31	0.451
Duration of anaesthesia (min)	53.6 ± 10.9	55.5 ± 11.2	0.345
Total sufentanil dosage (µg)	20.2 ± 2.64	20.7 ± 2.23	0.270
Remifentanil total dose (µg·kg^−1^)	7.79 ± 1.40	7.88 ± 1.44	0.724
Propofol total dose (mg·kg^−1^)	5.19 ± 0.88	5.23 ± 0.73	0.773
Preoperative CPR (mg·l^−1^)	0.70 (0.30—2.10)	0.75 (0.10—1.50)	0.706
Preoperative blood glucose (mmol·l^−1^)	4.93 ± 0.61	4.89 ± 0.65	0.742

Variables with Gaussian distribution were expressed as mean ± SD and analysed using the independent t-test, while variables with a non-Gaussian distribution expressed as a median and interquartile range and analysed using nonparametric test

ASA classifications and gender were expressed as number and analysed by Pearson χ2 test or Fisher exact test. Results showed both minimum expectation counts > 5 and n > 40, so we analysed them by Pearson chi-square test

*Abbreviations: BMI* Body mass index, *Group D* Dexamethasone group, *Group C* Control group

### Postoperative pain intensity and occurrence of painful event

**Fig. 3:**
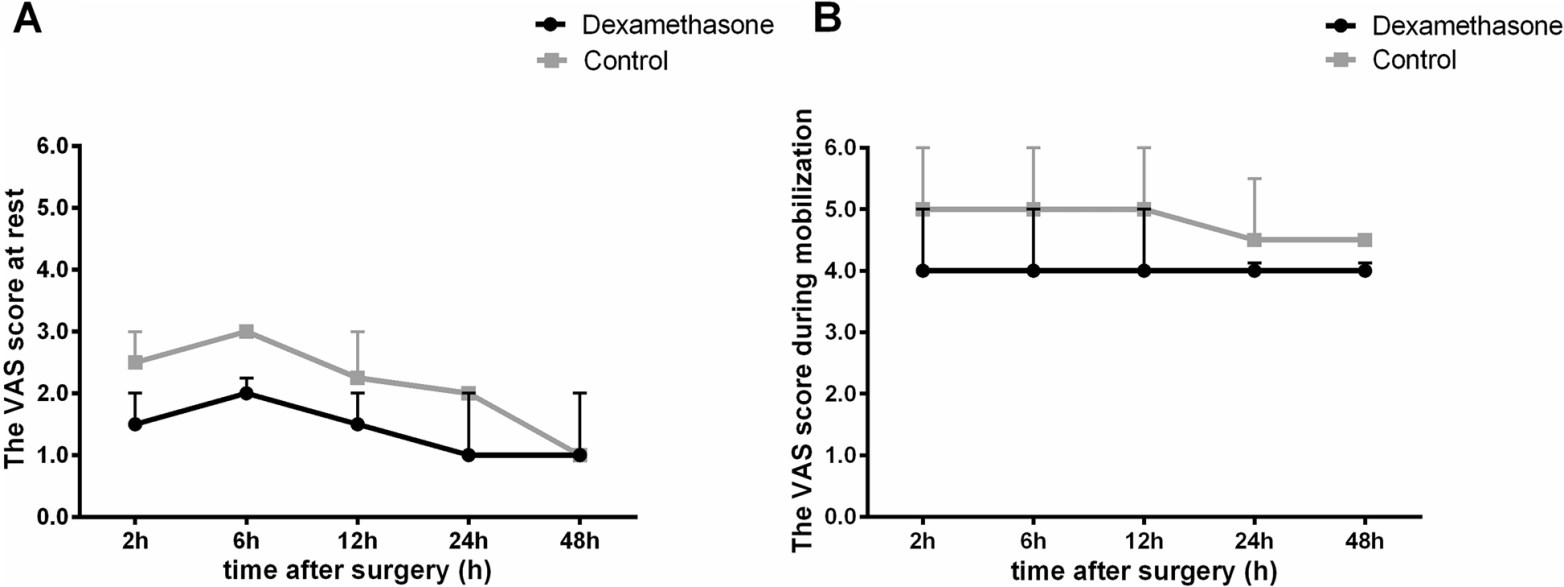
Comparison of postoperative VAS scores between the two groups. Values were expressed as as a median and interquartile range with error bars above, and analyzed with a linear mixed model analysis. We set the patient as the subject and the measurements at different time points postoperatively as the repetition factor, groups and postoperative time points were conducted as fixed effects while patient's intercept were conducted as the random effect. **A** The VAS score at rest in the two groups of patients during postoperative period. The degree of postoperative pain intensity reached the maximum at 6 h after operation, and then decreased slowly as time goes on. The analysis results showed that there was a significant statistical difference in postoperative pain between the two groups (*P* < 0.0001). **B** The VAS score during mobilization in the two groups of patients during postoperative period. LMM analysis showed that there was a significant statistical difference between the two groups (*P* < 0.0001). Abbreviations: VAS = Visual Analogue Scale; Group D, dexamethasone group; Group C, control group

The results showed that there were significant statistical differences between the two groups both at rest (F = 16.8, *P* < 0.0001) and during mobilization (F = 21.7, *P* < 0.0001), which indicated that patients in group D had significant lower postoperative pain scores than group C (Fig. 3).

Similarly, we calculated the percentage of occurrence of painful event (VAS score > 3) during 48 h after surgery and found that in group D was significantly lower than in group C both at rest {[0% (0%, 0%)] vs [0% (0%, 20%), *p* = 0.0014} and during mobilization {[80% (40%, 100%)] vs [100% (100%, 100%), *p* < 0.0001}.

### Facial swelling and trismus

Almost all patients had different degrees of facial swelling and limited mouth opening after surgery. Compared to Group C, patients in Group D had significantly light facial swelling and trismus at 24 h and 48 h postoperatively (*P* < 0.0125, Table 2 and Table 3). But there was no significant difference between the two groups at 6 h and 12 h after surgery (*P* > 0.0125).

**Table 2:** Incidence of postoperative facial swelling in two groups

		6 h post-op	12 h post-op	24 h post-op	48 h post-op
Group D (*n* = 58)	Light swelling	58(100%)	52(89.7%)	45(77.6%)	36(62.1%)
	Heavy swelling	0(0.00%)	6(10.3%)	13(22.4%)	22(37.9%)
Group C (*n* = 62)	Light swelling	62(100%)	49(79.0%)	19(30.6%)	14(22.6%)
	Heavy swelling	0(0.00%)	13(21.0%)	43(69.4%)	48(77.4%)
*P*-value		-	0.137	*P* < 0.0001	*P* < 0.0001

All values were expressed as number of patients (%) and analysed by Pearson χ^2^ test. *P* < 0.0125 indicated a statistical significance with a Bonferroni’s correction for 4 comparisons

*Abbreviations: Group D* Dexamethasone group, *Group C* Control group

**Table 3:** Incidence of postoperative trismus in two groups

		6 h post-op	12 h post-op	24 h post-op	48 h post-op
Group D (*n* = 58)	Light trismus	58(100%)	57(98.3%)	48(82.8%)	46(79.3%)
	Heavy trismus	0(0.00%)	1(1.70%)	10(17.2%)	12(20.7%)
Group C (*n* = 62)	Light trismus	62(100%)	55(88.7%)	31(50.0%)	28(45.2%)
	Heavy trismus	0(0.00%)	7(11.3%)	31(50.0%)	34(54.8%)
*P*-value		-	0.083	0.00022	0.00015

Values were expressed as number of patients (%). Values in 12 h post-op were analysed by Chi-square test of continuous correction, and other values were analysed by Pearson χ2 test. *P* < 0.0125 indicated a statistical significance with a Bonferroni’s correction for 4 comparisons

*Abbreviations: Group D* Dexamethasone group, *Group C* Control group

### Relevance of facial swelling and postoperative pain

There had a strong correlation between facial swelling and postoperative pain intensity both at rest and during mobilization at 6 h (*P* = 0.013 both), 12 h (*P* < 0.0001 both) and 24 h (*P* = 0.00078 and *P* = 0.00095) after surgery, but no statistical difference was shown between them at 48 h (*P* = 0.389 and *P* = 0.114) postoperatively (Table 4).

**Table 4:** Relevance of facial swelling and postoperative pain

		**At rest**				**During mobilization**			
		6 h post-op	12 h post-op	24 h post-op	48 h post-op	6 h post-op	12 h post-op	24 h post-op	48 h post-op
**FFacial swelling**	6 h post-op	0.225*				0.227*			
	12 h post-op		0.424**				0.362**		
	24 h post-op			0.303**				0.298**	
	48 h post-op				0.079				0.145

Values of postoperative pain and facial swelling were analysed by Spearman correlation test and values in the table were expressed with correlation coefficient

Bivariate correlation analysis, *P < 0.05, **P < 0.01

### CRP and blood glucose

**Fig. 4:**
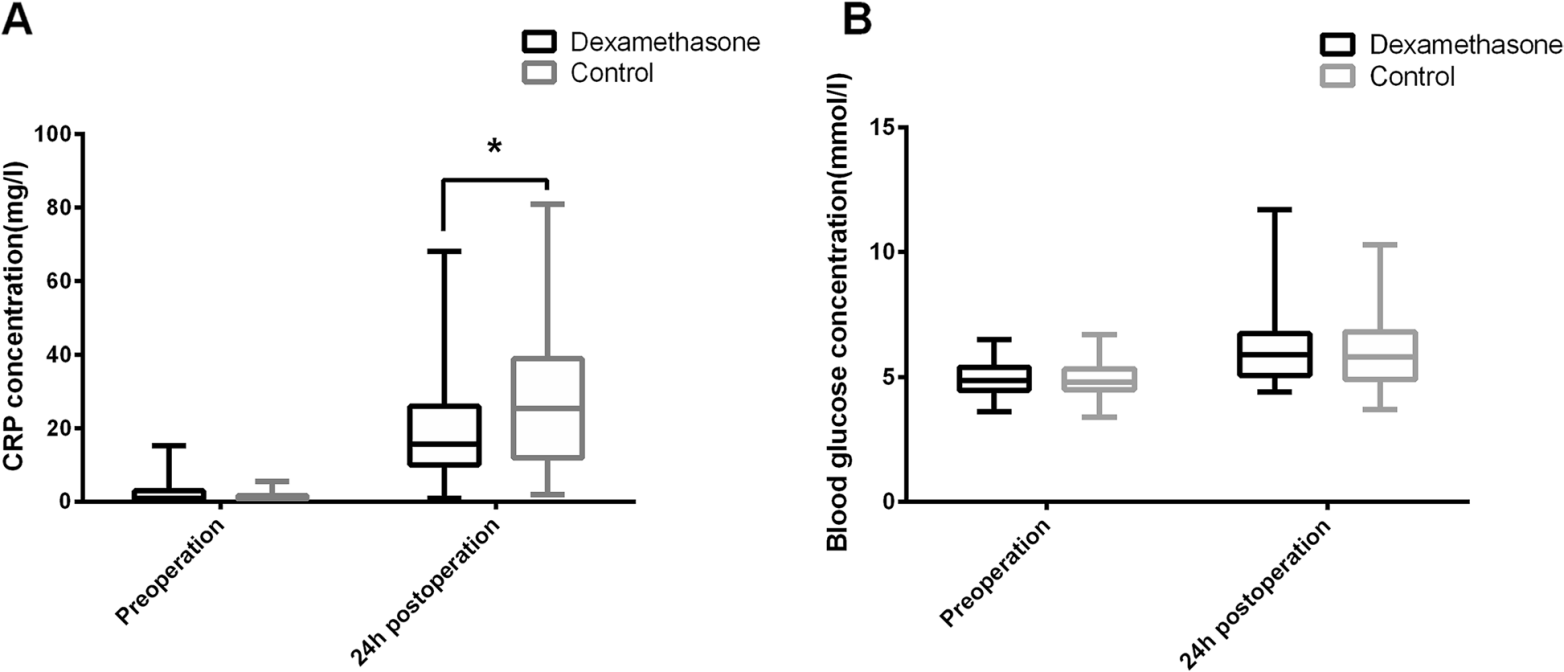
Comparison of CRP and blood glucose con between two groups. **A** Comparison of CRP concentration between two groups. Values were expressed as median and interquartile range and analyzed using Mann–Whitney U test between two groups. The CRP concentration after operation was significantly higher than preoperation in both groups (*P* < 0.05) by analyzed with the non-parametric paired Wilcoxon test. There was no difference in the preoperative CRP concentration between the two groups (*P* > 0.05), the CRP concentration after 24 h after surgery in group D was significantly lower than group C (*P* < 0.05). **B** Comparison of blood glucose concentration between two groups. The preoperative blood glucose concentration showed a Gaussian distribution with homogeneous variance, while the postoperative blood glucose concentration showed a non-Gaussian distribution. In order to ensure the consistency of the image format, all values in Fig. 4B are expressed as median and interquartile range, but the blood glucose of the two groups before operation was compared by independent sample t-test, while which after operation analyzed using Mann–Whitney U test. Compared with the preoperative measures, the postoperative blood glucose concentrations of both groups showed a significant increase and were statistically different (*P* < 0.05) by analyzed with the non-parametric paired Wilcoxon test. But there was no difference in the blood glucose concentration between two groups before and after surgery (*P* > 0.05). Note: Compared with group C, *P < 0.05; CRP = C-reactive protein

The level of CRP after surgery was significantly higher than preoperation in both groups [Group D: 15.6 (10.0–26.0) VS 0.70 (0.25–2.23), *P* < 0.0001; Group C: 25.3 (11.9–38.9) VS 0.75 (0.10–1.53), *P* < 0.0001]. There was no difference in the preoperative CRP concentration between the two groups {Group D [0.70 (0.25–2.23)] vs Group C [0.75 (0.10–1.53)], *P* = 0.706}, but after 24 h after surgery, the concentration of CRP in Group D [15.6 (10.0–26.0)] was significantly lower than in Group C [25.3 (11.9–38.9)] (*P* = 0.012, Fig. 4A).

Compared with the preoperative measures, the postoperative blood glucose concentrations of both groups showed a significant increase and were statistically different [group D: 5.90 (5.08–6.73) VS 4.85 (4.48–5.40), *P* < 0.0001; group C: 5.80 (4.90–6.80) VS 4.80 (4.50–5.33), *P* < 0.0001). But there was no difference in the blood glucose concentration between two groups in preoperation [Group D (4.93 ± 0.61) vs Group C (4.89 ± 0.65), *P* = 0.742, Table 1] and after surgery {Group D [5.90 (5.08–6.73)] vs Group C [5.80 (4.90–6.80), *P* = 0.608, Fig. 4B}.

## Discussion

This study showed that preoperative intravenous dexamethasone strengthened the analgesic effect of hydromorphone postoperatively and significantly alleviated postoperative pain, which is consistent with previous studies [10]. Despite there were a large number of related studies and conclusive results [11, 12], the mechanisms by which dexamethasone reduces postoperative pain were remain uncertain, that including immunosuppression [13], reduction of endotoxin levels [14] or gene regulation [15, 16], but most studies suggest that it is inextricably linked to its powerful anti-inflammatory effects [17, 18]. It was well known that microglia and various inflammatory factors which secreted played an important role in the generation and development of pain [19], and dexamethasone could alleviate pain by inhibit the activation and morphological changes of microglia from 0.5 h after tissue injury [20]. As our results showed, the pain intensity and occurrence of painful event in the treatment group decreased significantly from 2 h after operation (Fig. 3), which indicated that the patients with dexamethasone suffered much less pain intensity postoperatively.

Facial swelling and trismus are common postoperative complications of oral surgery. The external manifestations of swelling and difficulty in opening the mouth of the face are due to increased capillary permeability caused by persistent postoperative inflammation, which in turn leads to tissue and cellular oedema. A mate analysis by Falci [6] showed that dexamethasone had better prevention of postoperative complications than NSAIDs and was more effective than methylprednisolone in treating postoperative swelling and trismus. It was also in agreement with the results of our study (Table 2 and Table 3), that patients in the dexamethasone group had more light facial swelling and trismus than the control group from 24 h after the surgery, and the differences between the two groups became more pronounced at the time 48 h after operation as time went on. A research from Korea had found that the time point of maximum facial swelling appearence after oral surgery is in the 2.25 ± 0.19 days postoperative [21], so we could not find a significant difference between the two groups at 6 and 12 h after surgery as the time was too early to appear the difference. On the other hand, the pain intensity began to decrease in the time of 24 h post-operation for many surgeries [22], so we also couldn’t find the correlation between postoperative pain and facial swelling at 48 h after surgery, while our data confirmed the strong correlation between them both at rest and during mobilization at the other time points postoperatively according to the analysis of overall samples (Table 4).

C-reactive protein (CRP) was a sensitive index and marker reflecting various infectious and non-infectious systemic inflammation. In the study of knee arthritis and osteoarthritis models, it was found that the level of serum CRP was not only related to the development and prognosis of the disease, but also positively correlated with pain intensity [23, 24]. We further measured serum CRP concentrations in both groups and found that patients in the dexamethasone group had significantly lower CRP levels compared to the control group (Fig. 4A), a result that is consistent with the findings of Tammachote [25] and Kim [26], both of which demonstrate the great role of dexamethasone in inhibiting the production of postoperative inflammatory factors and the development of inflammation. Therefore, we believe that due to the development of postoperative inflammation forming facial swelling, which in turn further causes symptoms such as trismus, and these symptoms exacerbate the patient's post-operative pain and discomfort, but the process that is well suppressed by dexamethasone, as the development of post-operative inflammation and complications are inhibited, thus further reducing the development of postoperative pain.

The application of glucocorticoids was generally considered to bring side effects such as hyperglycemia and delayed surgical wound healing [27]. The results of our last study suggested that blood glucose concentrations were risen with different extent in both groups at 24 h after the operation, but there was no statistical difference between patients in the dexamethasone group and the control group (Fig. 4B), as a result, we consider more the occurrence of a stressful increase in blood glucose which was associated with surgery and anesthesia in the particular situation rather than the effect of dexamethasone [28, 29], which is consistent with the opinions of many research findings in domestic and international [30–32]. Therefore, we believe that there have evidence to support that perioperative intravenous treatment with 0.2 mg·kg^−1^ dexamethasone is safe in the perioperative period.

A limitation of our study is that we were unable to observe the patients' postoperative complications for a longer time due to the patients' hospitalization period, that made us uncertain about the clinical efficacy of dexamethasone for a prolonged time for postoperative complications after enucleation of jaw cysts. To improve and perfect the experiment and research, the next step of the study will be to set up regular post-operative follow-ups to prolong the observation of the development of postoperative complications in patients to get the best and most accurate clinical data.

## Conclusions

The mechanism of dexamethasone in preemptive analgesia may had multiple modes of action and there was no definite conclusion at present, but we believe that inflammation played an important role in the occurrence of postoperative pain in the perioperative period of oral surgery, dexamethasone could reduce the degree of facial swelling and trismus after the operation of curettage of jaw cyst by inhibiting inflammation to alleviate postoperative pain enormously, and had an excellent and safe efficacy in the clinical practice.

## Data Availability

The datasets generated and/or analyzed during the current study are not publicly available due to the data has not been completely uploaded in the database but are available from the corresponding author on reasonable request.

## Abbreviations

VAS: Visual analogue scale
ASA: American Society of Anesthesiologist
BP: Blood pressure
ECG: Electrocardiogram
BIS: Bispectral index
SD: Standard deviation
IQR: Interquartile range
CRP: C-reactive protein
SPI: Surgical Pleth Index
